# Alternative medicine and herbal remedies in the treatment of erectile dysfunction: A systematic review

**DOI:** 10.1080/2090598X.2021.1926753

**Published:** 2021-06-11

**Authors:** Kristian Leisegang, Renata Finelli

**Affiliations:** a School of Natural Medicine, Faculty of Community and Health Sciences, University of the Western Cape, Cape Town, South Africa; b American Center for Reproductive Medicine, Glickman Urological & Kidney Institute, Cleveland Clinic, Cleveland, OH, USA

**Keywords:** Erectile dysfunction, alternative medicine, herbal medicine, *panax ginseng*, *tribulus terrestris*, pygnogenol

## Abstract

**Objectives**: To systematically review and discuss the current evidence from placebo-controlled clinical trials that investigated the use of alternative medicines and herbal remedies in the management of erectile dysfunction (ED).

**Methods**: A Preferred Reporting Items for Systematic Reviews and Meta-Analyses (PRISMA)-based systematic review using specific keyword combinations was conducted on the PubMed and Scopus databases. Randomised controlled trials investigating herbal medicine in at least one group and using the International Index of Erectile Function (IIEF) as an outcome in patients primarily diagnosed with ED were included for review.

**Results**: Following the literature search, screening and eligibility analysis, a total of 42 articles were included. The 42 articles were categorised as single herb extractions (*n* = 14), combination herbal formula (*n* = 5), combination of herbal formula and non-herbal nutraceuticals (*n* = 7), non-herbal nutraceuticals (*n* = 5), acupuncture and moxibustion (*n* = 2), diet and nutrition (*n* = 3), exercise (*n* = 5), and topical treatments (*n* = 1). Based on the results, Korean ginseng, Pygnogenol and Prelox, *Tribulus terrestris, Lepidium meyenii*, L-arginine, acupuncture and lifestyle interventions were the more predominantly investigated treatments interventions for ED.

**Conclusions**: *Panax ginseng*, Pygnogenol, Prelox and *Tribulus terrestris* have promising evidence as herbal products, alongside L-arginine as a nutritional supplement, for ED based on IIEF outcomes, and warrant further clinical investigation. The mechanisms of action remain unclear, but each of these appears to in part increase nitric oxide synthesis. Importantly, improved diet and exercise should be considered, particularly in patients with obesity or diabetes mellitus.

## Introduction

Erectile dysfunction (ED) is defined as the inability to achieve and/or maintain an appropriate penile erection that is sufficient for sexual intercourse, and clinically classified as psychogenic (such as relationship dissatisfaction, societal pressures, anxiety or depression) or organic (underlying causes or comorbidities) [1,2]. This is a common and increasing male sexual health concern, with a prevalence of up to 31% [2] and projected to affect up to 322 million men by 2025 [3]. The prevalence of ED is reported as 1–10% of males aged <40 years (mostly psychogenic), >40% of men aged >40 years, and 50–100% of men aged >70 years (mostly organic) [3].

Importantly, ED has a significant negative impact on a man’s quality of life, including loss of self-esteem, avoidance of intimacy, anxiety and depression, which in turn exacerbate sexual dysfunction. This also impacts the relationship between sexual partners, including negatively affecting sexual desire and satisfaction of partners [4]. Furthermore, ED is considered an independent risk factor for the development of metabolic syndrome, cardiovascular disease and type 2 diabetes mellitus, sharing common underlying mediators, and therefore ED is considered an increased mortality risk [5–7]. As psychological, endocrine, immune and metabolic interactions underlie the pathogenesis [1], ED is considered a multidimensional and complex pathology [4].

The penis is comprised of arterioles and capillaries, blood filled sinuses, and smooth muscles. Sympathetic activation maintains smooth muscle contraction (vasoconstriction) and penile flaccidity [8]. Normal erection is initiated through external stimuli via somatic and autonomic pathways, where parasympathetic activation leads to nitric oxide (NO) production by the NO synthase (NOS) enzyme in endothelial cells and non-adrenergic non-cholinergic nerves in the penis [1,8]. Through molecular cascades, NO reduces cytosolic calcium, leading to smooth muscle relaxation and penile vasodilation (erection) [1,8].

The clinical assessment of ED requires a detailed history and clinical examination relevant to psychological and organic causes [1]. For diagnostic and research purposes, the diagnosis and severity of ED is classified based on the International Index of Erectile Function (IIEF) [9]. The IIEF is a validated subjective score with high sensitivity and specificity for ED [1], and is used as a global standard in the clinical investigation of conventional and surgical interventions for ED [9,10]. ED can be classified as severe (IIEF score ≤7), moderate (8–11), mild-to-moderate (12–16) and mild (17–21), while a IIEF score of ≥22 indicates no ED [9].

Pharmaceutical treatments for ED are typically phosphodiesterase-5 inhibitors that inhibit the outflow of blood from the penis to induce erection. Common examples include sildenafil citrate (Viagra), tadalafil (Cialis) and vardenafil (Levitra) [2,11]. Although generally well tolerated, adverse effects include headaches, dyspepsia, nasal congestion, flushing, syncope, vision loss, priapism, and myocardial infarction [12]. More invasive options for ED management include intracavernosal injection therapy of vaso-active substances, vacuum erection devices, and penile prosthesis implants [2,11]. However, not all patients respond to the treatments available, and adverse effects and costs may further limit pharmacological intervention [13]. These treatments also only deal with the symptoms of ED and do not address any underlying pathogenesis [14].

Complementary and alternative medicines, including dietary supplements and herbal remedies, are increasingly being used for treatment of ED, particularly through over-the-counter and internet sources [6,13,15,16]. These can be described as ‘a group of diverse medical and healthcare systems, practices, and products that are not presently considered to be part of conventional medicine’ [17,18]. This is a broad definition, incorporating traditional medicine systems and modalities. Examples include herbal medicines (remedies) and dietary supplements, nutritional and lifestyle therapies, acupuncture and traditional Chinese medicine, body therapies (such as massage, cupping and acupressure), homeopathy, mind–body techniques (such as meditation and yoga), energy medicine (such as reiki), and other traditional medicine disciplines such as Ayurveda, Unani and Naturopathy [18].

Up to 718 plant species are used in traditional medicine as aphrodisiacs, compounds that increase sexual arousal, libido, potency (erection) and/or sexual pleasure [13,19]. Top selling products on the market include items containing the herbs *Panax ginseng* (Korean ginseng), *Tribulus terrestris* (Tribulus), *Epimedium gradiflorum* (Horny goat weed), *Lepidium meyenii* (Maca), *Ginkgo biloba* (Ginkgo), *Eurycoma longifolia Jack* (Tongkat ali), and *Pausinystalia johimbe* (yohimbine), and nutraceuticals such as B complex vitamins, zinc, trace minerals, L-arginine, aspartate and dehydroepiandrosterone (DHEA) [6,15,20,21].

The increased use of alternative medicines and herbal remedies for ED with limited scientific investigations on extractions, efficacy, safety and dosage is a challenge to clinicians, whereas a comprehensive overview of clinically relevant research is these disciplines is currently lacking. Therefore, the present study aimed to systematically review and discuss the current evidence from placebo-controlled clinical trials that investigated the use of traditional medicine and herbal remedies in the management of ED as assessed by the IIEF.

## Methods

A systematic review of the literature for placebo-controlled clinical trials investigating traditional and herbal medicine was performed according to the Preferred Reporting Items for Systematic Reviews and Meta-Analyses (PRISMA) guidelines [22]. Studies of interest were placebo-controlled clinical trials investigating an alternative and/or herbal medicine in the treatment of ED that reported the IIEF as a primary outcome.

The keyword search was conducted on the PubMed and Scopus databases on 16 January 2021, using the following keyword string and combination of Boolean operators: (‘Phyto*’ OR ‘plant medicine*’ OR ‘polyherb*’ OR ‘herb*’ OR ‘natural medicine*’ OR ‘traditional medicine*’ OR ‘complementary medicine*’ OR ‘alternative medicine*’ OR ‘CAM’ OR ‘Chinese medicine’ OR ‘acupuncture’ OR ‘acupressure’ OR ‘moxibustion’ OR ‘tuina’ OR ‘qigong’ OR ‘hydrotherapy’ OR ‘homoeopathy’ OR ‘aromatherapy’ OR ‘aromatic oil*’ OR ‘nutraceutical*’ OR ‘supplement*’ OR ‘nutrition*’ OR ‘diet*’ OR ‘vitamin*’ OR ‘mineral*’ OR ‘reflexology’ OR ‘massage’ OR ‘exercise’ OR ‘unani’ OR ‘ayurveda’ OR ‘Asian medicine’ OR ‘eastern medicine’ OR ‘cupping’ OR ‘naturopathy’ OR ‘yoga’ OR ‘reiki’ OR ‘energy medicine’ OR ‘spiritual medicine’ OR ‘meditation’ OR ‘chiropractic’ OR ‘osteopathy’ OR ‘thermal therapy’ OR ‘light therapy’ OR ‘music therapy’ OR ‘Pycnogenol’ OR ‘Arginine’ OR ‘Aspartate’ OR ‘DHEA’ OR ‘Lepidium’ OR ‘Maca’ OR ‘Yohimbine’ OR ‘Epimedium’ OR ‘Ginseng’ OR ‘Ginkgo’ OR ‘Tongkat ali’ OR ‘Eurycoma’ OR ‘Tribulus’) AND (‘erectile dysfunction’ OR ‘IIEF’) AND (‘clinical’ OR ‘trial*’ OR ‘placebo’). The search was limited to English original articles only.

Articles retrieved were analysed for the removal of duplicates, and then screened based on the title and abstract. Subsequently, the full text was reviewed for eligibility based on the inclusion and exclusion criteria (Table 1). The retrieval, screening, and eligibility of articles for inclusion were conducted by two independent researchers (R.F., K.L.), and any disagreement was settled through discussion.

**Table 1.** Inclusion and exclusion criteria for eligibility

Inclusion criteria	Exclusion criteria
Randomised controlled trials in patients diagnosed with ED	Animal studies, *in vitro* studies, *in silico* studies
Sole use of traditional or herbal medicine as intervention in at least one group	Prospective and observational studies, case reports, meta-analysis and narrative reviews, conference abstracts and proceedings
Articles reporting the IIEF	Non-English language

## Results

**Figure 1.**
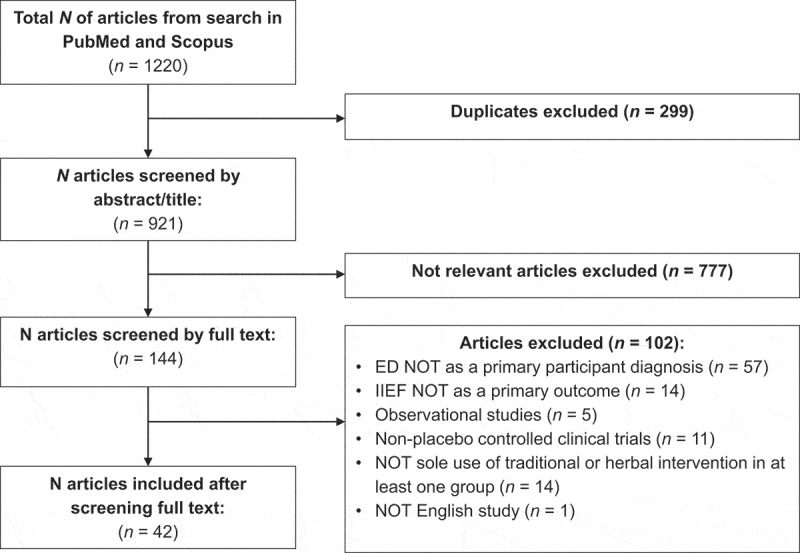
Flow diagram illustrating the methodology and results of the search strategy

The literature search retrieved a total of 1220 articles, of which 299 duplicates were removed. The remaining 921 articles were screened for suitability based on title and abstract, with 777 articles being excluded. A total of 144 full-text articles were then reviewed for eligibility based on the inclusion criteria (Table 1), and 102 articles were removed for not having ED as a primary participant diagnosis (*n* = 57) or the IIEF as a primary outcome (*n* = 14), for being observational studies (*n* = 5) or not placebo-controlled clinical trials (*n* = 11), for not sole use of traditional or herbal intervention in at least one group (*n* = 14), while one article was removed for non-English language. A total of 42 articles were included for the analysis and discussion (Figure 1).

Table 2 summarises the included studies [23–64]. These are categorised into single herb extractions (*n* = 14), combination herbal formula (*n* = 5), combination herbal formula and non-herbal nutraceuticals (*n* = 7), non-herbal nutraceuticals, (*n* = 5), acupuncture and moxibustion (*n* = 2), diet and nutrition (*n* = 3), exercise (*n* = 5), and topical treatments (*n* = 1).

**Table 2.** Alternative medicines and herbal remedies investigated in ED

Study design	Intervention dosage and duration	Patient cohort description (*n*)	Control group (*n*)		IIEF outcomes	Other outcomes	Adverse effects	Reference
Single herbal extractions								
Double-blind, randomised, placebo-controlled crossover trial	*Korean ginseng* (2700 mg/day for 4 weeks)	Patients with ED (45)	Placebo (45)		Significant increase in IIEF and the Erectile Function, Intercourse Satisfaction and Sexual Desire domains compared to control	Significant increase in percentage of rigidity of the tip of the penis compared to control; no significant difference in end-diastolic velocity, post-systolic velocity, and percentage of rigidity for the base of the penis compared to control	No adverse events reported by participants	23
Multicentre, double-blind, randomised, placebo-controlled trial	*Korean ginseng* berry extract (1400 mg/day for 8 weeks)	Mild-to-moderate ED (59)	Placebo (59)		Significant increase in IIEF-15 compared to control after 4 weeks; No significant increase in IIEF-15 compared to control after 8 weeks; Significant intragroup improvement for IIEF-15 after 4 and 8 weeks	No significant changes for total testosterone, HDL, LDL, and prolactin compared to control	No adverse events reported by participants	26
Double-blind, randomised, placebo-controlled trial	*Korean ginseng* (tissue-cultured mountain ginseng extract 2000 mg/day for 8 weeks)	Patients with ED (65)	Placebo (21)		Significant increase in IIEF and the Erectile Function, Intercourse Satisfaction and Overall Satisfaction domains compared to control	No significant changes for testosterone, LH, FSH, prolactin and oestradiol compared to control	Not reported by the authors	24
Double-blind, randomised, placebo-controlled trial	*Korean ginseng* (3000 mg/day for 12 weeks)	Patients with ED (30)	Placebo (30)		Significant increase in the IIEF-5 compared to control	No significant changes for testosterone, prolactin and cholesterol compared to control	Not reported by the authors	25
Double-blind, randomised, placebo-controlled trial	*Panax notoginseng* extract (1 capsule/day for 12 weeks)	Japanese adult men with low libido and IIEF scores (22)	Placebo (22)		Significant increase in the Intercourse Satisfaction of the IIEF compared to control	Significant increase in Androgen Deficiency in the Aging Male questionnaire, pre- and post-sleep penile circumference compared to control; No increase on serum PSA compared to control	No adverse events reported by participants	30
Double-blind, randomised, placebo-controlled trial	Pycnogenol (*Pinus pinaster* subsp. *Atlantica* 120 mg/day for 3 months	Patients with ED (21)	Placebo (21)		Significant increase in IIEF-5 compared to control	Significant increase of plasma antioxidant activity and significant decrease in total cholesterol and LDL compared to control	Not reported by the authors	31
Double-blind, randomised, placebo-controlled trial	Pycnogenol (*Pinus pinaster* subsp. *Atlantica* 120 mg/day for 4 months)	Patients with ED (32)	Placebo (21)		Significant increase in the Erectile Function domain of the IIEF compared to control	Significant reduction in total cholesterol and LDL compared to control; No significant change in HDL, triglycerides or glucose compared to control	No adverse events reported by participants	32
Double-blind, randomised, placebo-controlled trial	Tribestan (*Tribulus terrestris* herba extractum siccum (35–45:1) 1560 mg standardised to 675 mg furostanol saponins/day for 12 weeks)	Mild-to-moderate ED >6 months (86)	Placebo (86)		Significant increase in IIEF and the Orgasmic Function, Sexual Desire, Intercourse Satisfaction and Overall Satisfaction domains compared to control	Significant increase in Global Efficacy Question compared to control; No significant difference in total cholesterol, LDL, HDL, triglycerides, blood pressure, total and free testosterone, DHEA-S, and SHBG compared to control.	Reported as well tolerated with no significant difference between groups.	38
Double-blind, randomised, placebo-controlled trial	Trib Gold (*Tribulus terrestris*, standardised to contain 45% steroidal saponins, 750 mg/day for 3 months)	Ageing patients with ED and LUTS (35)	Placebo (35)		Significant increase in IIEF-5 compared to control	Significantly increased aspartate transaminase and PSA compared to control; No significant change in IPSS compared to control	Not reported by the authors	37
Double-blind, randomised, placebo-controlled trial	*Lepidium meyenii* (2400 mg/day for 12 weeks)	Mild ED (25)	Placebo (25)		Significant increase in IIEF-5 compared to control	Significant improvement in the Satisfaction Profile compared to control; No significant difference testosterone, FSH, LH and prolactin compared to control	No adverse events reported by participants	41
Double-blind, randomised, placebo-controlled crossover trial	Pomegranate juice (237 mL [8 fl oz] beverage/day for 4 weeks)	Mild-to-moderate ED (53)	Placebo (53)		No significant increase in IIEF-5 compared to control	Significant improvements in Global Assessment Questionnaires compared to control	Upper respiratory infections (8%), diarrhoea (2%), flatulence (2%), hyperlipidaemia (2%), nasal congestion (2%) and hypertension (2%)	56
Randomised, placebo-controlled trial	*Ashvattha Kshirpaka* (20 g powder of root, stem with bark, and budding leaves with 1 glass of milk/day for 45 days)	Diabetic and non-diabetic patients with ED (22)	Placebo (22)		Significant increase in IIEF and the Erectile Function, Intercourse Satisfaction and Orgasmic Function domains compared to control	No significant changes in total cholesterol, HDL, LDL, triglycerides, glucose, urea, creatinine, testosterone and DHEA-S compared to control	Not reported by the authors	57
Double-blind, randomised, placebo-controlled trial	*Butea superba* tubers (500 mg/day for 4 days followed by 1000 mg/day for a total of 3 months)	Patients with ED (17)	Placebo (14)		Significant intragroup increase in 4 of the 5 questions of the IIEF-5	No intragroup changes in haematology and blood chemistry analysis	Not reported by the authors	58
Double-blind, randomised, placebo-controlled trial	*Withania somnifera* (6000 g/day for 60 days)	Psychogenic ED (41)	Placebo (45)		No significant effect on IIEF-5 and IIEF-15 compared to control	None	Not reported by the authors	59
Combination herbal formula								
Multicentre single-blind, randomised, placebo-controlled trial	Tradamix TX1000 (Ecklonia bicyclis 600 mg), Tribulus terrestris 900 mg, and glucosamine oligosaccharide 500 mg daily for 3 months)	Mild-to-moderate ED (87)	Placebo (90)		Significant increase in IIEF and the Erectile Function, Orgasmic Function, Sexual Desire, Intercourse Satisfaction and Overall Satisfaction domains compared to control	Significant increase in the Male Sexual Health Questionnaire (Ejaculation Disorder), the Sexual Quality of Life scores and post-systolic velocity compared to control; No change in end-diastolic velocity or serum testosterone compared to control	No adverse events reported by participants	39
Multicentre Double-blind, randomised, placebo-controlled trial	VXP (*Panax ginseng* root 200 mg, *Serenoa repens* berry 200 mg, *Crategus rivularis* berry 200 mg, *Ginkgo biloba* leaf, *Turnera diffusa* leaf 200 mg, *Tribulus terrestris* vine 150 mg, *Erythroxylum catuaba* bark 100 mg, *Ptychopetalum olacoides* bark 100 mg, *Cuscuta chinensis* seed 50 mg, *Epimedium sagittatum* leaf 30 mg and Bioperine extract from *Piper nigrum* fruit 10 mg daily for 12 weeks)	Mild-to-moderate ED (39)	Placebo (36)		Significant increase in IIEF and the Erectile Function, Orgasmic Function, Sexual Desire, Intercourse Satisfaction and Overall Satisfaction domains compared to control	Significant increase in EDITS score compared to control; No significant change in testosterone, sperm count, semen volume, sperm motility, and investigator’s global assessment and subjects’ opinion compared to control	Well tolerated with minor adverse events not statistically different from placebo	29
Double-blind, randomised, placebo-controlled trial	Cappra® (*Cervus Nippon Temminck* 150 g, *Epimedium Drevicornum Maxim* 120 g, *Cynomorium Songaricum Rupr* 844 g, *Carthamus Tinctorius* 138 g, and *Cistanche Deserticola* 150 g daily for 2 weeks)	Mild-to-moderate ED (63)	Placebo (63)		Significant increase in IIEF and the Erectile Function, Orgasmic Function, Sexual Desire, Intercourse Satisfaction and Overall Satisfaction domains compared to control	No changes in haemoglobin, haematocrit, platelet count, alanine transaminase, aspartate transaminase, alkaline phosphatase, blood urea nitrogen, serum creatinine and glucose	Reported as well tolerated; minor reports included dizziness (13.3%), face numbness (1.6%), and tachycardia (1.6%)	60
Double-blind, randomised, placebo-controlled trial	NRL/MW/201,901 (L-citrulline and extracts of *Withania somnifera, Mucuna Pruriens, Anacyclus pyrethrum, Abutilon indicum, Trigonella foenum-qraecum, Ginkqo biloba, Myristica fragrans, Pansx ginseog, Tribulus terrestris* and *Svzygium aromaticum* daily for 60 days)	Mild-to-moderate ED (50)	Placebo (36)		Significant increase in IIEF and the Erectile Function, Orgasmic Function, Sexual Desire, Intercourse Satisfaction and Overall Satisfaction domains compared to control	Significant increase in Quality of Erection questionnaire, number of sexual encounters, intra-vaginal ejaculation latency time and serum testosterone compared to control	No adverse effects reported by participants	40
Preliminary randomised, placebo-controlled trial	KBMSI-2 (*Ginseng Radix Rubra, Dioscorea tenui-pes, Cornus officinalis Sieb. et Zucc., Lycium chinense Mill*, and *Curcuma longa Linn –* 12 g/day for 6 weeks)	Patients with ED (19)	Placebo (20)		Significant increase in IIEF, Erectile Function and Intercourse Satisfaction domains compared to control	No significant changes in Aging Males’ Symptoms Scale and serum total testosterone compared to control	One mild adverse event reported	28
Combination herbal formula and non-herbal nutraceuticals								
Double-blind, randomised, placebo-controlled crossover trial	Prelox (L-arginine aspartate 3 g and Pycnogenol 80 mg daily for 1 month)	Mild-to-moderate ED (50)	Placebo (50)		Significant increase in IIEF-15 compared to control	Significant increase in sperm intracellular e-NOS and serum testosterone compared to control	No adverse events reported by participants	33
Double-blind, randomised, placebo-controlled trial	Prelox (L-arginine aspartate 2.8 g and Pycnogenol 80 mg daily for 6 months)	Mild-to-moderate ED (54)	Placebo (57)		Significant increase in IIEF and the Erectile Function, Orgasmic Function, Sexual Desire, Intercourse Satisfaction and Overall Satisfaction domains compared to control	Significant increase serum testosterone compared to control; No significant effects on blood pressure, cholesterol or glucose compared to control	Excellent risk profile with few adverse effects reported by participants	34
Double-blind, randomised, placebo-controlled trial	L- arginine (690 mg), aspartic acid (552 mg) and Pycnogenol (Prelox) (60 mg) daily for 2 months	Mild-to-moderate ED (11)	Placebo (12)		Significant increase in IIEF and the Intercourse Satisfaction domain compared to control	Significant decrease in blood pressure, aspartate transaminase and γ‐glutamyl transpeptidase, and increase in salivary testosterone compared to control	No adverse events reported by participants	36
Double-blind, randomised, placebo-controlled crossover trial	Pycnogenol® (80 mg), roburins, L-arginine, L-citrulline (1 month)	Moderate ED (25)	Placebo (25)		Significant increase in IIEF and Erectile Function domain compared to control	None	Without unwanted side-effects	35
Double-blind, randomised, placebo-controlled trial	SX (L-arginine glutamate and yohimbine (daily for 4 weeks)	Mild-to-moderate ED (20)	Placebo (20)		Significant improvement in Erectile Function domain compared to control	None	Adverse events not statistically different from placebo	44
Double-blind, randomised, placebo-controlled three-way crossover trial	L-arginine glutamate (6 g) and yohimbine hydrochloride (6 mg) daily for 2 weeks	Mild-to-moderate ED >3 months (48)	Placebo (48)		Significant increase in IIEF and Erectile Function, Intercourse Satisfaction and Overall Satisfaction domains compared to control	None	Well tolerate and few adverse events. Minor adverse events included headache and insomnia	43
	Yohimbine hydrochloride (6 mg) daily for 2 weeks				No significant increase in IIEF compared to control; Significant intragroup improvement for IIEF			
Double-blind, randomised, placebo-controlled trial	Vitamin E (100 IU), *Korean ginseng* (67 mg) and *Eleutherococcus senticosus* (40 mg) daily for 6 weeks	Patients with ED (26)	Placebo (26)		Significant increase in Erectile Function domain of IIEF compared to control	None	No clinically important adverse effects reported	27
Non-herbal nutraceuticals								
Double-blind, randomised, placebo-controlled trial	L-arginine (5 g) daily for 4 weeks	Type 2 diabetics with mild-to-moderate ED (40)		Placebo (26)	Significant increase IIEF and the Erectile Function, Orgasmic Function, Sexual Desire, Intercourse Satisfaction and Overall Satisfaction domains compared to control	Significant increase in testosterone compared to control	Not reported by the authors	42
Double-blind, randomised, placebo-controlled crossover trial	L-arginine aspartate (8 g) and adenosine monophosphate (200 mg) taken 1–2 h before intercourse	Mild-to-moderate ED (26)		Placebo (26)	Significant increase in overall IIEF, the Erectile Function and Intercourse Satisfaction domains compared to control	Significant increase in Erection Hardness Score and EDITS score compared to control	Reported as well tolerated; minor GIT complaints reported in 2 patients	44
Double-blind, randomised, placebo-controlled trial	Myoinositol (4 mg) and folic acid (400 µg) daily for 12 weeks	Type 2 diabetes with ED >6 months (88)		Placebo (88)	Significant intragroup increase in IIEF-5	Significant intragroup increase in end-diastolic velocity and post-systolic velocity compared to control	Not reported by the authors	61
Double-blind, randomised, placebo-controlled trial	Niacin 1500 mg/day for 12 weeks	ED with hyperlipidaemia (61)		Placebo (65)	Significant intragroup increase in the IIEF and Erectile Function domain compared to control	Significant intragroup increase in the Sexual Health Inventory for Men	Patients reported flushing (36.3%), itchiness (32.5%), headache (5%), gastric discomfort (3.8%), palpitations (3.8%), presyncope (2.5%), chest pain (1.3%) and others (10%). Flushing and itchiness were reported significantly more compared to control	62
Double-blind, randomised, placebo-controlled trial	DHEA 50 mg/day for 24 weeks	Patients with ED (17)		Placebo (13)	Significant increase in IIEF and the Erectile Function, Orgasmic Function, Sexual Desire, Intercourse Satisfaction and Overall Satisfaction domains compared to control	Significant increase in DHEA and testosterone compared to control; No significant change in prolactin, PSA, prostate volume and post-void residual volumes compared to control	No adverse effects reported by participants	63
Acupuncture and moxibustion								
Randomised, placebo-controlled trial	Acupuncture specific against ED (unreported duration)	Psychogenic ED (19)	Acupuncture specific against headache (1)		Significant intragroup increase in overall IIEF, Erectile Function and Intercourse Satisfaction domains	None	Not reported by the authors	46
Randomised, placebo-controlled trial	Acupuncture with warm needling moxibustion (4 sessions)	Patients with ED (24)	Conventional acupuncture without warm needling moxibustion (22)		Significant increase in IIEF-5 compared to control	None	Not reported by the authors	47
Lifestyle (diet and nutrition)								
Single-blind, randomised, controlled trial	Tailed caloric intake reduction and physical activity increase advice to achieve a loss of ≥10% in their total body weight for 2 years	Obese males with ED without diabetes, hypertension, or hyperlipidaemia (55)		General information about healthy food choices and exercise (55)	Significant increase in IIEF-5 compared to control	Significant decrease in BMI, cholesterol, triglycerides, glucose, insulin, IL6, IL8 and CRP compared to control	Not reported by the authors	48
Single-blind, randomised controlled trial	Detailed advice about how to reduce body weight, improve quality of diet, and increase physical activity for 2 years	Male patients with ED (104)		General information about healthy food choices and exercise (105)	Significant increase in IIEF-5 compared to control	Significant decrease in BMI, waist circumference, blood pressure, glucose and insulin, and increase in HDL, compared to control; no significant change in total cholesterol and insulin	Not reported by the authors	49
Parallel feeding, randomised, controlled trial	60 g/day of a mixture of raw walnuts, almonds, and hazelnuts for 14 weeks	Males with ED (43)		Usual Western-style diet avoiding nuts (40)	Significant increase in the Orgasmic Function and Sexual Desire domains of the IIEF compared to control	No difference in serum NO and E-selectin compared to control	No adverse events reported by participants	50
Lifestyle (exercise)								
Randomised controlled trial	Interval exercise training programme (8 weeks of 60–79% heart rate max reserve for 45–60 min/day)	Hypertensive patients with ED (21)	Age-matched sedentary hypertensive control group (21)		Significant increase in IIEF compared to control	Significant decrease in serum CRP compared to control	Not reported by the authors	52
Randomised controlled trial	Aerobic physical activity (150 min of moderate intensity aerobic activity/week for 3 months)	Vascular ED (50)	Age-matched patients with vascular ED who did not accept the physical activity (50)		Significant increase in IIEF-5 compared to control	Significant increase in peak systolic velocity and significant decrease in acceleration time compared to control; significantly lower serum concentrations of original immunophenotype endothelial progenitor cells and endothelial microparticles compared to control	Not reported by the authors	51
Randomised controlled crossover trial	Pelvic floor muscle exercises enhanced by manometric biofeedback and lifestyle changes (reducing alcohol consumption, stopping smoking, reducing weight, getting fit and avoiding bicycle saddle pressure) daily for 3 months	ED >6 months (28)	Lifestyle changes (27)		Significant intragroup increase in IIEF-5	Significant intragroup improvement in men with post-micturition dribble	Not reported by the authors	54
Randomised controlled crossover trial	Pelvic floor muscle exercises enhanced by manometric biofeedback and lifestyle changes	ED >6 months (28)	Lifestyle changes (27)		Significant increase in the Erectile Function domain of IIEF compared to control	Significant improvement in anal pressure and digital anal grades compared to control	Not reported by the authors	53
Randomised controlled trial	Sexual rehabilitation (physical exercise training, pelvic floor exercise and psychoeducation for 4 months)	ED with ischaemic heart disease or implantable cardioverter defibrillator (77)	Usual care (77)		Significant increase in IIEF-5 compared to control	Significant increase exercise capacity and pelvic floor strength compared to control; no significant difference in the Psychosocial Adjustment to Illness Scale compared to control	Not reported by the authors	55
Topical treatments								
Double-blind, randomised, placebo-controlled trial	*Crocus sativus* gel (pea-sized amount on half the penis 30 min before intercourse for 1 month)	Diabetic men with ED (25)	Placebo (25)		Significant increase in IIEF and the Erectile Function, Orgasmic Function, Sexual Desire, Intercourse Satisfaction and Overall Satisfaction domains compared to control	None	Not reported by the authors	64

BMI: body mass index; CRP: C-reactive protein; EDITS: Erectile Dysfunction Inventory of Treatment Satisfaction; GIT: gastrointestinal tract; HDL: high-density lipoprotein; IL: interleukin; LDL: low-density lipoprotein; SHBG: sex hormone-binding globulin.

## Discussion

The results of the systematic review reveal a paucity of studies for many alternative medicines and herbal remedies used for ED. A total of 42 studies were retrieved, which generally varied significantly on the interventions used to investigate ED in controlled trials. This included single and combined herbal interventions, non-herbal nutraceuticals, combined herbal and non-herbal nutraceuticals, acupuncture, lifestyle intervention, and topical herbal applications. However, based on the results and related literature, Korean ginseng, Pygnogenol and Prelox, *Tribulus terrestris, Lepidium meyenii*, L-arginine, acupuncture and lifestyle interventions are further discussed in detail.

### Panax ginseng

In traditional Chinese medicine practice, *Panax ginseng* (Korean ginseng), particularly the steamed aged root called red ginseng, has been used as an aphrodisiac to improve sexual performance for thousands of years [65,66]. Indeed, *Panax ginseng* has become a popular global herbal supplement for male reproductive disorders, including sexual performance and ED [67]. The present review included four studies investigating *Panax ginseng* based on IIEF outcomes, where three reported benefit [23–25] and one reported no change [26]. An additional study combined ginseng with vitamin E, which also improved erectile function [27]. Ginseng was further used in a combination herbal formula including KBMSI-2 [28] and VigRX Plus (VXP) [29], both improving erectile function. Furthermore, one study was found to investigate the related species *Panax notoginseng*, finding an improvement in Intercourse Satisfaction only, although there was an improvement in the Androgen Deficiency in the Aging Male questionnaire, pre- and post-sleep penile circumference [30].

These results agree with previously published Korean language placebo-controlled trials, where ginseng significantly improved erectile function and sexual satisfaction reports by patient and partner [68–70], as well as penile blood flow [71]. However, Kim *et al*. [72] reported no benefit of *Panax ginseng* in a placebo-controlled trial that was based on the Watts Sexual Function questionnaire. Based on a meta-analysis of five clinical studies, Borrelli *et al*. [73] also concluded that *Panax ginseng* significantly improved erectile function.

Ginsenosides (steroid-like saponins) are considered the major active isolates that are unique to ginseng species, particularly *Panax ginseng* [74]. These are a heterogeneous group of triterpenoidal glycosides that vary based on the number of sugars and the bonding positions on the aglycone skeleton [75]. The plant also contains polysaccharides, alkaloids, and phenolic compounds [76]. Importantly, the bioactivity of ginseng requires both saponin and non-saponin metabolites working synergistically [76]. Ginseng extractions and ginsenosides have been shown in animal studies to induce NO synthesis in the endothelium, inducing vasodilation of the corpus cavernosum and subsequent erection [65]. Ginseng is also reported to increase testosterone concentrations, improving erection and increasing copulatory behaviour [65]. However, few studies included in the results of the present systematic review reported a positive impact of ginseng on testosterone [24–26], neither did the combination formulae containing ginseng [28,29]. Borelli *et al*. [73] also reported no pooled increase in testosterone with ginseng supplementation.

*Panax ginseng* is further considered safe, without significant herb–drug interaction at dosages of 0.5–3 g/day [74]. However, significantly more research on the potential mechanisms and active isolates for ginseng to improve ED is warranted.

### Pygnogenol and Prelox

*Pinus pinaster* Ait. subsp. *Atlantica* (Maritime pine) is a tree native to Southwestern France. Pygnogenol is a proprietary patented extraction of the powdered *Pinus pinaster* bark, standardised to 70% ± 5% procyanidins [77,78]. Procyanidins are biopolymers of catechin and epicatechin subunits, with significant antioxidant properties that have numerous health promoting and disease management benefits [79]. Pygnogenol has significant antioxidant, anti-inflammatory and immune regulating properties [80–85]. Importantly, evidence further suggests increased vascular NO synthesis and vasodilation, and catecholamine antagonists action, which can improve erection [78,86].

Pygnogenol was reported in two studies as a sole herbal intervention for ED, with both studies reporting improvement in erectile function [31,32]. Prelox is a combination of Pygnogenol®, L-arginine, L-citrulline and roburins, included as a combination therapy in four studies that all reported a benefit in erectile function and other domains of the IIEF across the studies [33–36]. Prelox also reported improved testosterone levels [33,34,36]. However, the impact of arginine with Pygnogenol is not clear. In an uncontrolled trial, L-arginine caused a non-significant improvement in erectile function over 1 month. Following the introduction of Pygnogenol alongside L-arginine in the same cohort caused a significant improvement of erectile function over the next month. In the third month, doubling the dose of Pygnogenol further improved the normal erection in the cohort [87]. This suggests that Pygnogenol may be effective without the inclusion of arginine in the form of Prelox. Pygnogenol therefore appears to be supported by these clinical trials for use in ED. However, more research is warranted to establish efficacy, dosage and mechanisms of action of Pygnogenol in ED.

### Tribulus terrestris

*Tribulus terrestris* is a medicinal plant used for thousands of years in India and China, and well documented in the Ayurvedic, Unani, Siddha and Chinese Traditional Medicine systems [88]. Alongside ginseng, it is a common herb used in male sexual health products [67]. Steroidal saponins spirostanol and furostanol are characteristic secondary metabolites of *Tribulus terrestris*, along with flavonoids. Alkaloids include those related to β-carboline and amide alkaloids [89].

*Tribulus terrestris* was investigated as a single herb in two of the included studies, both using standardised extractions for saponins and reporting a positive effect on the IIEF and various domains [37,38]. Furthermore, with *Tribulus terrestris* as the major constituent, the combination formulae Tradamix TX1000, VXP, and NRL/MW/201,901 were also shown to increase IIEF and all domains [29,39,40]. This generally agrees with the review by Borrelli *et al*. [73], although a non-English study excluded from this review found no difference to placebo in IIEF or testosterone [90].

Animal studies suggest a pro-erectile effect of *Tribulus terrestris* on the corpus cavernosum. This is mediated through increased NO in the endothelium and nitrergic nerve endings, inducing vasodilation. Animals also show a dose-dependent increase in sexual behaviour [88,89]. Furthermore, *Tribulus terrestris* is suggested to increase testosterone levels [88,89]. However, there was no increase reported by the three studies in the present review that included testosterone analysis [29,38,39]. Further studies are needed to determine efficacy and potential mechanisms of *Tribulus terrestris* for ED.

### Lepidium meyenii

*Lepidium meyenii* (Maca or Peruvian bark), a member of the Brassicaceae family, is native to the Central Andes Mountains of Peru at altitudes of 4000–4500 m. As a food supplement and for medicinal purposes, this plant has been used traditionally for >2000 years [91,92]. Important and unique secondary metabolites identified include imidazole alkaloids, hydantoins, meyeniins A − C, alkamides, glucosinolates, and phytosterols [92].

Only one study included in the present review investigated *Lepidium meyenii* as a single herbal intervention, improving IIEF with no significant impact on testosterone [41]. This agrees with the review by Borrelli *et al*. [73]. In a systematic review investigating *Lepidium meyenii* on male and female sexual function [93], an additional two studies were further reported in healthy male cohorts that both showed no significant effect on sexual function compared to placebo [94]. In the Shin *et al*. [93] review, it was concluded that *Lepidium meyenii* improves sexual desire over a period of at least 6 weeks.

Animal studies investigating sexual behaviour in rats using pulverised *Lepidium meyenii* hypocotyls have yielded mixed results [91]. Here, glucosinolates are proposed as being active in male sexual function [92]. Although sexual function is regulated partly by testosterone, there is little evidence that *Lepidium meyenii* can increase serum or testicular testosterone [91,94,95]. However, animal studies have further suggested improvement of ED through increase in the intracavernosal pressure to mean arterial pressure ratio [92].

Although reported as well tolerated in the included study, *Lepidium meyenii* may cause psychological adverse events, including anxiety, mood swings, hallucinations, and addictive behaviour [15]. Significantly more clinical and laboratory research is warranted to determine efficacy, dosage, and tolerability.

### L-arginine

L-arginine is an amino acid obtained through dietary sources, particularly meats and nuts, or produced from L-citrulline [96]. Importantly, L-arginine is the only substrate for NOS to produce NO [97]. Through vasodilation mediated by increased NO, L-arginine has been found to be effective in hypertension [97]. This vasodilation also mediates increased cavernosal blood flow [67]. The excellent bioavailability profile of L-arginine further demonstrates a positive effect on NO production [96].

L-arginine was found to be used as a sole intervention in one study, where a significant improvement was reported [42]. There were four studies combined with Pycnogenol as Prelox that all reported positive effect on the IIEF [33–36]. L-arginine was combined with yohimbine hydrochloride in two studies, also both reporting a positive impact on the IIEF [43,44]. Lastly, L-arginine was also found to improve the IIEF in combination with adenosine monophosphate [45].

In a systematic review, Chang Rhim *et al*. [98] included a total of 10 studies, with four studies using L-arginine as a sole therapy. Of those 10 studies, six were included in the results of the present systematic review [33,34,42–45]. In two excluded studies that used the Cologne Erectile Inventory (KEED) as an outcome, L-arginine improved sexual dysfunction, alongside ED [99] and O’Learys Questionnaires [100]. In healthy Japanese male cohorts without ED, L-arginine improved the IIEF when combined with citrulline [101] and ornithine [102]. Pygnogenol, yohimbine, adenosine monophosphate and ornithine may be used in combination as they are all proposed to improve NO, and therefore may offer synergistic effects [96]. L-arginine may further be more effective in patients with ED with low NO, where patients with low urinary nitrites and nitrates appear to have greater benefit [100]. This dietary supplement was reported as well tolerated by all studies. As a natural dietary supplement with good absorption and bioavailability, this can allow for long duration of treatment for ED [96].

### Acupuncture

Acupuncture is an ancient and traditional Chinese medicine practice established for over 2500 years that has become popular globally over recent decades [103]. It is defined as the insertion of needles into specific points of the body that can be further manipulated using various techniques. The proposed mechanism is to manipulate meridians, which can simply be described as energy channels, to restore energy (*qi*) balance between *yin* (female, moon, dark, concealed) and *yan*g (masculine, bright, open) in the body to restore health [103,104]. Acupuncture research can be difficult to bridge ancient and modern principles, where there are some barriers to research that include basic terminology, the difficulties in standardised treatment methods, and effective measures for assessing treatment [105]. However, acupuncture has been investigated in various forms of reproductive medicine [18].

Acupuncture treatment was reported to be beneficial in one included study in the results [46]. With the addition of warm needling moxibustion, it was also shown to be superior to standard acupuncture [47]. Both studies are relatively small and not clearly reported. This broadly agrees with the systematic review by Cui *et al*. [106], where too few studies are available for any conclusions for the efficacy of acupuncture in ED [2].

### Lifestyle changes

Lifestyle changes are part of almost all traditional systems of medicine, although these may vary on the traditional basis of the advice provided [18]. There were a total of eight studies that investigated nutritional and/or exercise in male cohorts with ED. Two studies by the same group investigated specific nutritional advice and exercise in obese [48] and non-obese male patients with ED over a 2-year duration [49]. This improved the IIEF compared to controls who just received general information on healthy lifestyles, alongside improvement of numerous metabolic parameters.

This is supported by the Mediterranean diet showing clinical benefit to reduce the deterioration of sexual function in type 2 diabetics in both males and females compared to those on a low-fat diet, where systemic inflammation predicted ED and severity [107]. The Mediterranean diet further reduced prevalence of ED in men with metabolic syndrome, alongside inflammatory markers and endothelial function scores [108]. This diet is rich in seeds and nuts, olive oil, seafood, whole grains and fruits and vegetables [108]. In this context, the intervention of nuts (raw walnuts, almonds, and hazelnuts) over 2 weeks improved the Orgasmic Function and Sexual Desire domains of the IIEF compared to a standard Western diet devoid of nuts [50]. Furthermore, dietary based weight loss in obese and diabetic men also improved sexual and endothelial function [109,110], where a high protein, moderate carbohydrate and low-fat diet further improved systemic inflammation [110]. Inflammation, particularly through tumour necrosis factor-alpha, reduces NOS expression, which inhibits vasodilation. Importantly, this is mediated through intracellular reactive oxygen species generation in the endothelial cells [111]. The improvement in inflammatory markers may partly explain the mechanism of appropriate nutrition on erectile function, particularly in obese and diabetic men. Here, a healthy nutritional approach may improve NO production with a correlated reduction in pro-inflammatory cytokines, which can improve ED in men with metabolic syndrome [112].

The association between ED and cardiovascular diseases is mediated in part by endothelial dysfunction, in which poor nutrition and sedentary lifestyle are contributing factors to the shared risk [113]. Regular physical activity can improve sexual dysfunction and ED through multiple proposed mechanisms that are relevant to ED and cardiovascular disease, including endocrine modulation, increased NO, improved arterial blood pressure, lipoprotein and glucose regulation [114]. Specific aerobic training [51] and interval exercise programmes [52] were shown to be effective in two of the included studies. These interventions further improved inflammation [52] and vascular function [51] in these patients. This is supported by Khoo *et al*. [114], reporting that moderate intensity and high volume exercise of >200 min/week improve weight, waist circumference, sexual function and testosterone compared to small exercise volume in abdominally obese and sedentary males. Furthermore, specific pelvic floor exercises were found to be effective in improving the IIEF compared to lifestyle changes in two studies [53,54]. Pelvic floor exercises combined with physical activity and psychoeducation for sexual rehabilitation also reported an improvement in erectile function in males with ischaemic heart disease or implantable cardioverter defibrillator [55].

Nutritional and exercise-based approaches appear to be beneficial for ED. This is more apparent in obese, metabolic syndrome and diabetic men. Therefore, the integration of appropriate nutrition and exercise programmes should be considered as part of a holistic approach to ED.

## Limitations and strengths

The participants in the present included studies were varied, across different forms of ED including psychogenic or organic, as well as comorbidities such as obesity, hypertension, dyslipidaemia and diabetes, which limits any comparison between studies. In many studies, the underlying pathogenesis or mechanisms of action were not investigated. Although standardised and validated, the IIEF remains a subjective scoresheet that has some limitations. Furthermore, the present review did not consider a single intervention, and numerous different forms of alternative medicines and herbal remedies are reported. Lastly, only 42 studies were included, which is a small representation of the alternative medicines and herbal remedies used for ED, making any conclusions relatively unclear even for the more prominent interventions investigated.

These limitations were partially addressed by using a keywords search, which was expansive for various common modalities of alternative medicines and herbal remedies. This was supported by the inclusion of common alternative medicines and herbal remedies used on the market to capture further studies. The PRISMA guidelines were used as a framework for the systematic review, while the inclusion of studies only with placebo/no treatment comparison groups and standardised and validated clinical outcome (IIEF) provided some consistency in the review.

## Conclusion

The reported use of alternative medicines and herbal remedies for ED is extensive, particularly through dietary supplements available on the market. However, there is limited research into specific treatments in the improvement of erectile function. There is also a significant amount of heterogeneity in the interventions investigated and the dosage and duration. Based on the present results, *Panax ginseng*, Pygnogenol or Prelox and *Tribulus terrestris* have some promising evidence as herbal products, alongside L-arginine as a nutritional supplement. The mechanisms of action remain unclear, but each of these appears to increase NO synthesis and NO production to induce erection. Importantly, improved diet and exercise should be considered, particularly in patients with obesity or diabetes mellitus.

## Abbreviations

: DHEA: dehydroepiandrosterone; ED: erectile dysfunction; IIEF, International Index of Erectile Function; NO(S): nitric oxide (synthase); PRISMA: Preferred Reporting Items for Systematic Reviews and Meta-Analyses; VXP: VigRX Plus
